# GOT2: New therapeutic target in pancreatic cancer

**DOI:** 10.1016/j.gendis.2024.101370

**Published:** 2024-07-02

**Authors:** Jiarui Bu, Zeyu Miao, Qing Yang

**Affiliations:** Department of Pathogenobiology, College of Basic Medical Sciences, Jilin University, Changchun, Jilin 130021, China

**Keywords:** Glutamine metabolism, GOT2, Pancreatic cancer, PPARδ, Tumor microenvironment

## Abstract

In recent years, the incidence and mortality rates of pancreatic cancer have been steadily increasing, and conventional therapies have shown a high degree of tolerance. Therefore, the search for new therapeutic targets remains a key issue in current research. Mitochondrial glutamic-oxaloacetic transaminase 2 (GOT2) is an important component of the malate-aspartate shuttle system, which plays an important role in the maintenance of cellular redox balance and amino acid metabolism, and has the potential to become a promising target for anti-cancer therapy. In this paper, we will elaborate on the metabolic and immune effects of GOT2 in pancreatic cancer based on existing studies, with a view to opening up new avenues for the treatment of pancreatic cancer.

## Introduction

Pancreatic cancer is a common malignant tumor in the digestive system. Existing data indicate that the incidence of pancreatic cancer has been increasing in recent years, while its 5-year overall survival rate has been decreasing, and it is expected to become the second leading cause of cancer-related death by 2030.^1^^,^^2^ In terms of treatment, due to its insensitivity to most chemotherapeutic agents and poor therapeutic response, surgical resection is currently the only curative method for pancreatic cancer. Unfortunately, pancreatic cancer has an insidious onset and inconspicuous clinical symptoms. 80%–85% of patients are diagnosed at an advanced stage that is difficult to resect, and there is a high recurrence rate even after surgical treatment.^3^ With the continuous deepening of research in recent years, metabolic therapy and immunotherapy have gradually entered people's vision. The research on metabolic or immune targets of pancreatic cancer may provide new ideas for its clinical treatment.

Glutamic-oxaloacetic transaminases (GOTs), also known as aspartate transaminases, play a crucial role in amino acid metabolism and the tricarboxylic acid (TCA) cycle and can be classified into two subtypes according to their subcellular localization, namely GOT1 in the cytoplasm and GOT2 in the mitochondria. In normal physiology, GOTs play key roles in cellular energy metabolism. Firstly, GOTs participate in glutamine metabolism and mediate the interconversion of glutamate and oxaloacetate with α-ketoglutarate and aspartate by catalyzing transamination reaction, the products of which directly promote cell proliferation. For example, aspartate (Asp) serves as a precursor for the synthesis of many amino acids and provides raw materials for protein and nucleotide synthesis,^4^^,^^5^ while α-ketoglutarate (α-KG) plays a critical role in the production of ATP and the replenishment of intermediates in the TCA cycle.^6^ Secondly, GOTs are key components of the malate-aspartate shuttle system,^7^ which enable NADH to transfer between the cytoplasm and mitochondria, and maintain cellular redox balance. Recent studies have revealed that apart from its traditional metabolic functions, there is also an unconventional function of GOT2 in the nucleus, which may be achieved through fatty acid binding and uptake.^8^ Based on the above key roles of GOT2 in cells, researchers have deduced that GOT2 may be a potential therapeutic target for various cancers and have initiated relevant studies.

**Figure 1:**
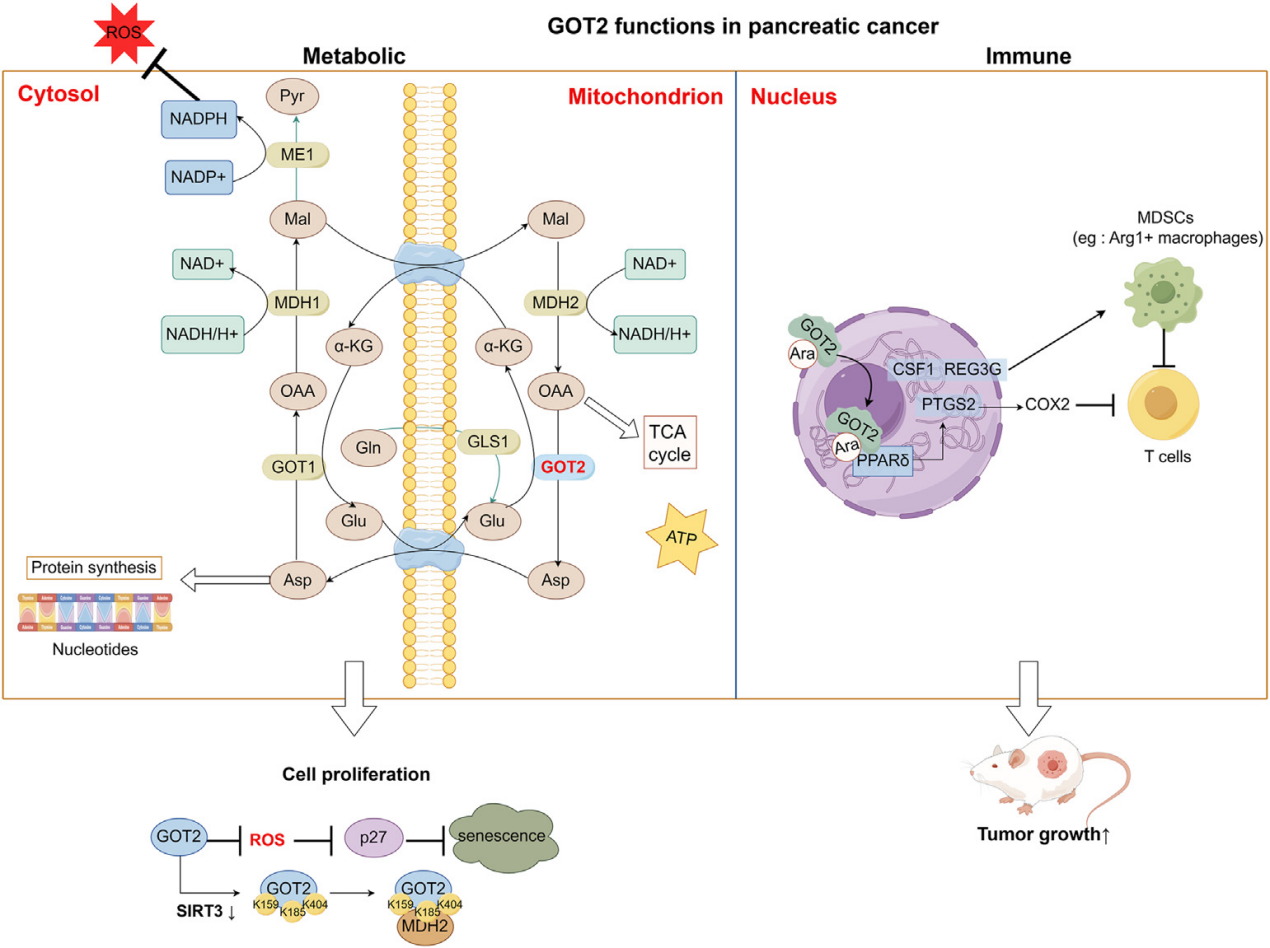
Metabolic and immune functions of GOT2 in pancreatic cancer cells. First, GOT2 catalyzes transamination reaction in mitochondria, and the products can directly promote cell proliferation. Aspartate provides raw materials for protein and nucleotide synthesis, and oxaloacetate participates in the TCA cycle. Second, GOT2 is a key component of the malate-aspartate shuttle system, which maintains cellular redox balance. In addition, pancreatic cancer cells exhibit unique glutamine metabolism. On this basis, decreased SIRT3 expression leads to an increased level of GOT2 acetylation. The acetylation of GOT2 at 3K sites enhances the protein association of GOT2-MDH2, which promotes the malate-aspartate NADH shuttle activity to increase ATP production and stimulates NADPH production to inhibit ROS, thereby reducing the expression of cyclin-dependent kinase inhibitor p27 and suppressing cellular senescence (leftmost plot). An unexpected nuclear role of GOT2 in pancreatic cancer: As a nuclear fatty acid transporter, GOT2 promotes the transcriptional activity of PPARδ by directly binding to fatty acids (mainly arachidonic acid) and induces the expression of immunomodulatory target genes. PTGS2 encodes COX2 that inhibits T cell-mediated anti-tumor immunity. CSF1 and REG3G encodes secretory protein which recruits myeloid-derived suppressor cells (MDSCs) into the tumor microenvironment (right plot). OAA, oxaloacetate; α-KG, α-ketoglutarate; Ara, arachidonic acid; Asp, aspartate; COX2, cyclooxygenase 2; CSF1, colony stimulating factor 1; Glu, glutamine; Gln, glutamine; GOT2, glutamic-oxaloacetic transaminase 2; Mal, malate; MDH2, malate dehydrogenase 2; PPARδ, peroxisome proliferator-activated receptor δ; PTGS2, prostaglandin endoperoxide synthase 2; Pyr, pyruvate; REG3G, regenerating islet-derived protein 3 gamma; ROS, reactive oxygen species; SIRT3, sirtuin-3; TCA, tricarboxylic acid. This picture was drawn by Figdraw.

In this paper, we will comprehensively and systematically elaborate on the metabolic and immune functions of GOT2 and the corresponding molecular mechanisms in pancreatic cancer based on existing research (Fig. 1), with the aim of providing new ideas and approaches for pancreatic cancer treatment.

## Metabolic roles of GOT2 in pancreatic cancer

The occurrence and development of tumors depend on cellular metabolic reprogramming.^9^^,^^10^ Metabolic therapy focuses on the metabolic dependence of cancer cells.^11^ Therefore, it is essential to fully understand how metabolism is regulated in tumor cells. Pancreatic cancer cells are strongly dependent on unique glutamine metabolism, which utilizes glutamine (Gln)-derived Asp to maintain cell redox state.^12^ Targeting specific metabolic enzymes in this pathway may bring new hope for pancreatic cancer treatment.

Enzymes involved in the glutamine metabolic reprogramming (mitochondrial GLS1 (glutaminase 1), GOT1, and GOT2) are highly up-regulated in pancreatic cancer.^13^ In mitochondria, Gln is catabolized to glutamate via GLS1 and further converted into TCA cycle intermediates α-KG and Asp via GOT2, providing raw materials for mitochondrial energy metabolism. Next, Asp is transported to the cytoplasm and converted to oxaloacetate via GOT1, and then to malate, which is finally catalyzed by malic enzyme 1 (ME1) to generate pyruvate and produce NADPH to inhibit reactive oxygen species (ROS) generated during rapid cell proliferation and maintain cellular redox homeostasis.^12^ This metabolic reprogramming of pancreatic cancer is mainly mediated by mutant KRAS (KRAS∗), and that oncogenic KRAS^∗^ is the signature genetic event for pancreatic cancer progression.^14^^,^^15^ Non-canonical glutamine metabolism is crucial for pancreatic cancer growth. Targeting this pathway has become a new approach for pancreatic cancer treatment.^16^, ^17^, ^18^

GOT2 plays a critical role as a component of glutamine metabolic reprogramming. Currently, studies have demonstrated that GOT2 depletion leads to impaired proliferation and disrupted redox homeostasis in pancreatic cancer cells *in vitro*.^19^ Consistent with its conventional metabolic effects, GOT2 knockdown (KD) in cultured pancreatic cancer cells results in decreased Asp and α-KG production, thereby preventing the normal TCA cycle and decreasing ATP levels. This suggests that GOT2 promotes glutamine anaplerosis. Second, GOT2 KD forces the malate-aspartate shuttle to interrupt and prevents the transfer of NADH from the cytoplasm to the mitochondria, which induces cellular NADH accumulation and reductive stress, and results in the block of glyceraldehyde 3-phosphate dehydrogenase (GADPH) node and impaired glycolytic function. Meanwhile, loss of GOT2 leads to a decrease in cellular NADPH/NADP^+^ ratio and a significant increase in ROS levels,^20^ which could be aggravated under hypoxic conditions.^21^

Existing studies have explored this pathway to regulate redox homeostasis to obtain more effective treatment strategies for pancreatic cancer. GOT2 can inhibit pancreatic cancer cell senescence by maintaining cellular redox balance.^20^ Here, GOT2 KD induces the expression of the cyclin-dependent kinase inhibitor p27 by increasing ROS levels in the cells and triggers its mediated cellular senescence. Relevant literature suggests that p27 is down-regulated in several human cancers and plays a critical role in regulating cell cycle progression and inducing and maintaining senescence.^22^^,^^23^ Since KRAS^∗^ and combinations of mutation in p53 and p16^24^ may allow pancreatic cancer cells to bypass classical senescence pathways, targeting senescence pathways regulated by GOT2 may be important in overcoming therapeutic resistance in pancreatic cancer. In addition, SIRT3 (sirtuin-3)-dependent GOT2 acetylation stimulates the malate-aspartate NADH shuttle activity and oxidative protection, thereby promoting pancreatic tumor growth.^25^ GOT2 acetylation at lysine residues K159, K185, and K404 (3K), enhances the protein association between GOT2 and malate dehydrogenase 2 (MDH2), thereby facilitating malate-aspartate shuttle activity. SIRT3 has been identified as the main deacetylase of GOT2, and its deacetylation may not affect the enzyme activity of GOT2 itself but impairs the GOT2-MDH2 association, which negatively regulates malate-aspartate shuttle and impairs pancreatic cancer cell proliferation. Thus, SIRT3 may act as a tumor suppressor in pancreatic cancer,^26^ and GOT2 deacetylation drugs targeting this need to be explored. Given the elevated levels of GOT2 K159 acetylation in pancreatic cancer, this could be a potential biomarker for pancreatic cancer diagnosis.^25^

Notably, GOT2 is essential for maintaining growth and inhibiting senescence in pancreatic cancer, but has little effect on proliferation and senescence in untransformed human pancreatic ductal cells, which may be related to the greater dependence of normal cells on glutamate dehydrogenase 1 (GLUD1).^12^^,^^20^ Therefore, targeting GOT2 may provide sufficient therapeutic window to protect the surrounding tissues from damage and become one of the effective and specific strategies for treating pancreatic cancer. Impaired Gln metabolism has been found to cause pancreatic cancer cells to be more sensitive to oxidative stress,^12^ which may have synergy with treatments that increase cellular ROS levels, such as radiotherapy and chemotherapy. In addition, recently developed targeted drugs, such as KRAS^∗^ selective inhibitors^27^^,^^28^ and GLS inhibitors,^13^^,^^29^^,^^30^ are in various stages of preclinical or clinical trials and are considered to have important value in pancreatic cancer treatment, but still have limitations such as resistance or weak efficacy of single therapy. So GOT2-targeted therapy may be an ideal combination or alternative approach and is expected to have a profound impact on future treatment.

Overall, GOT2 as a hub for glutamine metabolic reprogramming contributes to cancer progression by providing Asp and α-KG for biosynthesis and energy requirements. In fact, various cancers show sensitivity to glutamine deprivation.^16^ However, GOT2 has different regulatory mechanisms in different tumors,^31^, ^32^, ^33^, ^34^, ^35^ giving us a new perspective on how GOT2-mediated glutamine metabolism functions in cancers. BRCA1/ZBRK1 repressor complex reduces Asp biosynthesis through transcriptional repression of GOT2 expression, thereby leading to impaired proliferation of breast cancer cells.^31^ In non-small cell lung cancer, miRNA down-regulates GOT2 at post-transcriptional level. Silencing circ-SEC31A significantly affects malate-aspartate metabolism, and inhibits cell proliferation, migration, and invasion by regulating the miR-520a-5p/GOT2 axis.^32^ Importantly, although hepatocellular carcinoma (HCC) similarly exhibits glutamine addiction, GOT2 is lowly expressed in HCC cells, which is related to advanced progression and poor prognosis.^33^ Here, GOT2 KD maintains glutathione/ROS balance by facilitating glutathione synthesis, thereby activating the PI3K/AKT/mTOR pathway and ultimately promoting HCC progression. GOT2 plays a suppressive and promoting role in HCC and pancreatic cancer, respectively, which may be associated with generating glutathione through different pathways to maintain cellular redox balance.

## Immune role of GOT2 in pancreatic cancer

Recently, a study has revealed an unexpected nuclear role of GOT2 in pancreatic cancer. Distinct from its traditional metabolic function, GOT2 acts as a nuclear fatty acid transporter to promote the transcriptional activity of peroxisome proliferator-activated receptor δ (PPARδ) by directly binding to fatty acids and inhibit T cell-mediated anti-tumor immunity, thereby facilitating pancreatic cancer progression.^8^

Abrego et al first assessed the significance of GOT2 on pancreatic cancer progression and found GOT2 knockdown had little or no impact on the proliferation of pancreatic cancer cells *in vitro*. Similarly, in immunodeficient mouse transplantation models, tumor growth did not change after silencing GOT2. However, tumor growth was severely impaired in immune-functioning syngeneic mice. Consistent with the results *in vitro*, tumor cell proliferation *in vivo* was not impaired. These results suggest that the effect of GOT2 on tumor growth *in vivo* may be immune-mediated. In accordance with this view, GOT2-silenced tumor cells showed an increase in T-cell content, including CD4^+^ and CD8^+^ T cells, and a decrease in immunosuppressive Arg1^+^ macrophage abundance. After treatment with neutralizing antibodies against T cells, GOT2-silenced tumor growth was restored. The authors further found that GOT2 was localized in the nucleus in pancreatic cancer cells and acted as a fatty acid-binding protein to regulate the transport of nuclear fatty acids, and bind to and activate PPARδ, promoting its transcriptional activity. PPARδ undergoes conformational changes after binding to nuclear fatty acids, altering its binding to DNA and inducing the expression of target genes.^36^ Prostaglandin endoperoxide synthase 2 (PTGS2) is one of the immunomodulatory genes regulated by PPARδ and encodes COX2 (cyclooxygenase 2) which induces prostaglandin synthesis. Recent reports have revealed that COX2 contributes to the reduction of T-cell infiltration in the pancreatic cancer microenvironment^37^ and is associated with anti-tumor immunosuppression in other cancers.^38^ In addition, other genes activated by PPARδ, such as CSF1 (colony stimulating factor 1) and REG3G (regenerating islet-derived protein 3 gamma), encode secreted proteins that recruit myeloid-derived suppressor cells to the tumor microenvironment. Furthermore, arachidonic acid has been identified as the major GOT2-binding fatty acid mediating the GOT2-PPARδ interaction.

The study of Abrego and colleagues has revealed the part-time function of the metabolic enzyme GOT2 in pancreatic cancer: the GOT2-PPARδ axis significantly regulates the immune microenvironment of pancreatic cancer to inhibit anti-tumor immunity. This provides new insights into pancreatic cancer treatment and prompts us to consider whether targeting GOT2 to promote immune response could extend beyond pancreatic cancer cells. Recent studies have revealed the direct roles of GOT2 in immune cells.^39^, ^40^, ^41^ In preclinical models, co-expression of exogenous GOT2 enhances the metabolism and proliferative capacity of anti-GPC3 CAR-T cells and significantly increases the anti-tumor activity of CAR-T cells in specific solid tumors.^39^ The importance of GOT2 in T cells is due to its metabolic roles. Another study showed that GOT2 plays an important role in the metabolic reprogramming of diffuse large B-cell lymphoma.^40^ GOT2 provides Asp and nucleotides to cells with activated or abnormal Jak/STAT and NF-κB signaling pathways, thereby promoting cell proliferation. In addition, GOT2 and GLS as prognostic biomarkers for breast cancer, are closely associated with dendritic cells and immunotherapy response.^41^ Cluster 2 (lower GOT2 expression and higher GLS expression) shows a better immunotherapy response. In short, these findings suggest the possibility of establishing a close link between glutamine metabolism and immunity for cancer treatment, which allows us to overcome tumor immune evasion and adapt immunotherapies to improve patient outcomes.

## Alternative mechanisms by which pancreatic cancer cells bypass GOT2 silencing

**Figure 2:**
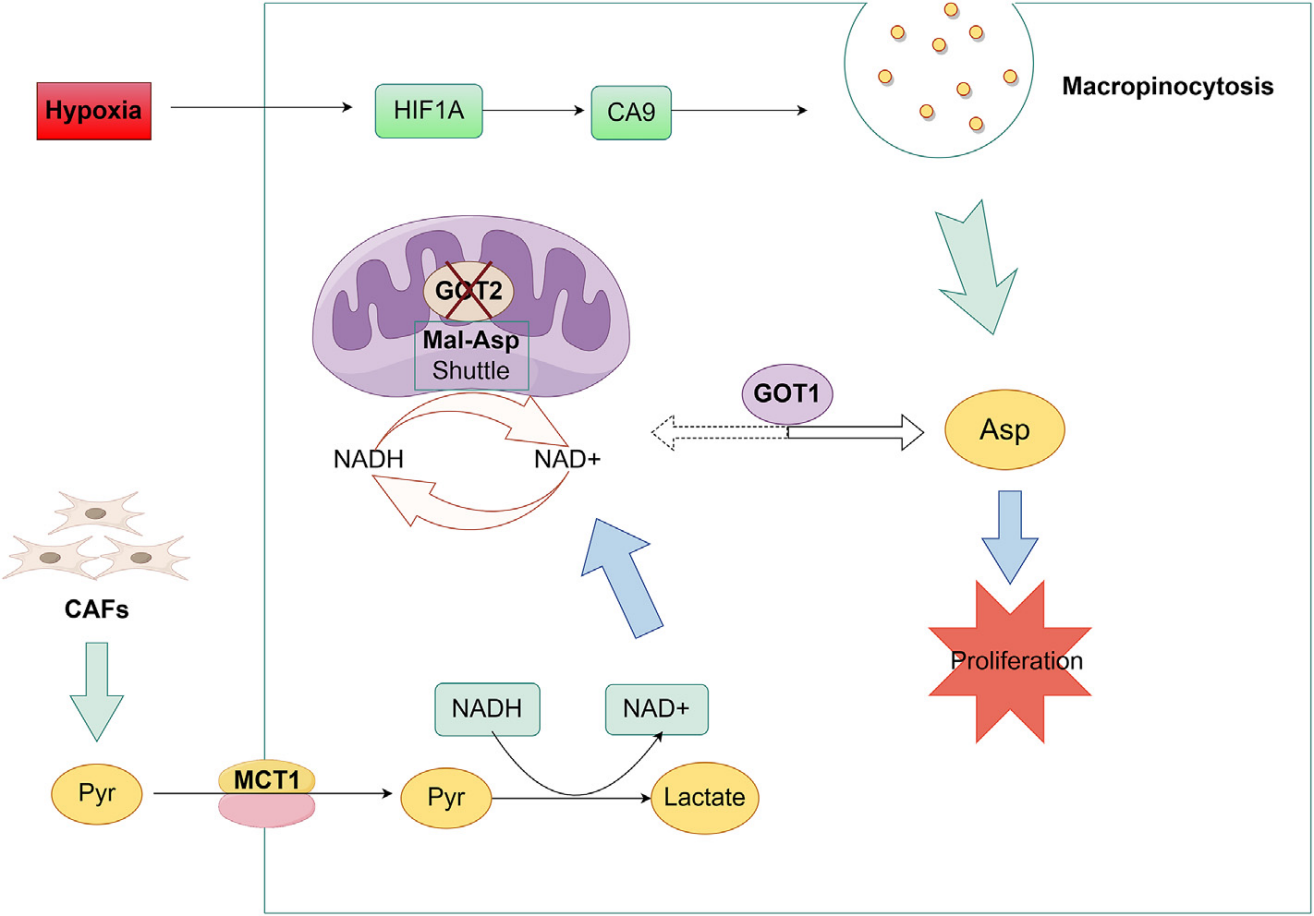
Alternative mechanisms bypass GOT2 silencing in pancreatic cancer cells. CAFs can secrete pyruvate in the pancreatic tumor microenvironment. Here pyruvate is transported to pancreatic cancer cells via MCT1 and converted into lactate, which can restore the redox imbalance caused by GOT2 loss and reverse the GOT1 pathway to synthesize Asp, thus saving pancreatic cancer cell proliferation *in vitro*. Under hypoxia conditions, HIF1A enhances the effect of macropinocytosis by inducing CA9 expression. Macropinocytosis can directly provide enough Asp to GOT2-deficient pancreatic cancer cells by capturing environmental albumin and releasing amino acids inside the cells. These two alternative routes for acquiring Asp allow pancreatic cancer to bypass the metabolic limitations from GOT2 loss. Asp, aspartate; CA9, carbonic anhydrase IX; CAFs, cancer-associated fibroblasts; GOT1/2, glutamic-oxaloacetic transaminase 1/2; HIF1A, hypoxia-inducible factor 1 subunit α; MCT1, monocarboxylate transporter 1; Pyr, pyruvate. This picture was drawn by Figdraw.

Pancreatic cancer exhibits a unique tumor microenvironment.^42^^,^^43^ Due to the poor vascular function of pancreatic tumors, pancreatic cancer cells are exposed to a long-term hypoxic microenvironment and severely restricted in terms of nutrients,^44^ which damages the ability to transfer electrons to oxygen in the mitochondria, thereby limiting Asp synthesis.^4^^,^^5^ Under hypoxic conditions, pancreatic cancer cells depend on GOT2 to synthesize endogenous Asp.^21^^,^^45^ However, pancreatic tumors *in vivo* may use alternative pathways to obtain Asp, thereby bypassing the dependence on GOT2. Two recent studies have elucidated the compensatory mechanisms of pancreatic cancer cells to overcome GOT2 loss *in vivo*, based on cell extrinsic and cell intrinsic factors (Fig. 2).^19^, ^21^

Kerk et al found that GOT2 deficiency impaired pancreatic cancer cell proliferation *in vitro* but had no effect on tumor growth *in vivo*.^19^ Given that pancreatic cancer exhibits a unique GOT2-dependent glutamine metabolism, there must be other compensatory metabolic pathways for pancreatic cancer cells *in vivo* to survive under GOT2-deficient conditions. Cancer-associated fibroblasts (CAFs) are major components of the pancreatic tumor microenvironment,^46^ such as human pancreatic stellate cells. CAFs indirectly compensate for the insufficient Asp due to GOT2 deficiency by releasing the redox-active metabolite pyruvate to GOT2-deficient pancreatic cancer cells. Here, pyruvate, as an electron acceptor, is transported into pancreatic cancer cells via monocarboxylate transporter 1 (MCT1) and converted to lactate via lactate dehydrogenase (LDH), which can restore the redox imbalance caused by GOT2 deficiency and reverse the GOT1 pathway to synthesize Asp. CAFs conditioned medium can rescue the proliferation of GOT2-deficient pancreatic cancer cells *in vitro*, while inhibiting pyruvate uptake and metabolism can block their recovery. However, these interventions are ineffective against GOT2 KD tumors *in vivo*, which may indicate the presence of other Asp sources in pancreatic tumor microenvironment.

Macropinocytosis is a lysosomal degradation pathway driven by pancreatic cancer cells expressing KRAS^∗^, which can provide enough Asp to support pancreatic cancer cell to survive in nutritional constraints by capturing environmental albumin and releasing amino acids within the cells.^47^, ^48^, ^49^ Thus, this nutrient scavenging pathway leads to pancreatic cancer resistance to therapies targeting non-classical metabolic pathways. Garcia-Bermudez et al revealed a relationship between hypoxia and macropinocytosis.^21^ Macropinocytosis supports the proliferation of hypoxic pancreatic cancer cells, and hypoxia in turn enhances the effect of macropinocytosis. Here, hypoxia-inducible factor 1 subunit α (HIF1A) induces carbonic anhydrase IX (CA9) expression^50^ and increases the production of its catalytic product bicarbonate, thereby up-regulating micropinocytosis. Currently, inhibitors targeting scavenging pathway have made progress in preclinical studies or clinical trials in pancreatic cancer, including lysosome acidification inhibitors^51^^,^^52^ and CA9 inhibitors.^53^ New and more potent autophagy inhibitors are also being developed,^54^^,^^55^ which may block pancreatic cancer cells from acquiring Asp via micropinocytosis and hopefully slow the growth of GOT2 KD pancreatic tumors.

The above two studies suggest that pancreatic cancer cells interact with the tumor microenvironment to open up alternative routes to acquire Asp, allowing pancreatic cancer to bypass the metabolic constraints from GOT2 deficiency. In the complex tumor microenvironment of pancreatic cancer, in addition to the metabolism of pancreatic cancer cells themselves, the metabolism between stromal cells and immune cells can also regulate tumor progression.^10^ Therefore, it is important to consider the role of tumor microenvironment in metabolic therapies targeting GOT2 in pancreatic cancer and gain a better understanding of its potential resistance mechanisms, which will be beneficial to develop more effective metabolic therapies.

## Conclusion

According to existing research, we have a deeper understanding of the roles of GOT2 in pancreatic cancer, and in addition to its well-known metabolic significance, the unexpected role of immunosuppression also deserves our attention. Cellular metabolic reprogramming and intrinsic immune escape are key features of pancreatic cancer, and GOT2, as a metabolic enzyme, bridges organically the two, presenting a broader prospect for targeting GOT2 to treat pancreatic cancer.

Small molecule inhibitors of GOT2 are expected to be effective therapeutics for pancreatic cancer. Studies have shown that the pan-transaminase inhibitor amino oxyacetate (AOA) is a promising strategy for GOT2-targeted cancer therapy. By inhibiting malate-aspartate shuttle, AOA can damage the pathway of glucose conversion to TCA cycle products, thereby inhibiting the proliferation of breast cancer cells *in vitro* and tumor growth in xenograft models of athymic mice.^56^ AOA treatment selectively induced the death of glutamine-dependent *MYC*-amplified glioblastoma cell line *in vitro* without affecting the viability of Myc-deficient cell line.^57^ In addition, recent studies have identified several GOT1 inhibitors^58^, ^59^, ^60^ that may provide new ideas for potential strategies to target GOT2. As a KAT2 inhibitor, PF-04859989 inhibits GOT1 activity in a time- and pyridoxal-5 ′-phosphate (PLP)-dependent manner and selectively impairs pancreatic cancer cell line growth. Unfortunately, the inhibitory activity of PF-04859989 on GOT2 is relatively low.^58^ Therefore, the development of potent GOT2 inhibitors is a challenging next step.

Before these findings can be applied to the clinic, some important issues remain to be resolved. First, the selection of preclinical models may cause differences in study results. Kerk et al^19^ and Garcia-Bermudez et al^21^ found that proliferation of pancreatic cancer cells *in vitro* is dependent on GOT2, but that GOT2 is not required for tumor growth in immunodeficient or immunocompetent mouse xenograft models of pancreatic cancer. However, Abrego et al obtained the exact opposite results.^8^ GOT2 knockout did not affect the proliferation of pancreatic cancer cells *in vitro* and tumor progression in immunocompromised mouse models, but severely impaired xenograft growth in mice with intact immune systems. This prompts us to develop mouse models that more accurately reproduce human disease to fully understand how the metabolic and immune effects of GOT2 interact in pancreatic cancer. Secondly, targeting the GOT2-PPARδ axis may have great therapeutic potential. This requires us to further clarify the regulatory mechanisms of GOT2 nuclear localization and the precise molecular mechanisms by which GOT2 regulates PPARδ transcriptional activity. Moreover, recent studies have revealed compensatory mechanisms of pancreatic cancer to bypass GOT2 dependence,^19^^,^^21^ which prompts us to target resistance mechanisms for improved treatment and to focus on whether fibroblasts mediate resistance to GOT2 silencing in the intact immune system. Finally, because pancreatic cancer patients suffer from severe immunosuppression and dysfunction of T-cell populations,^61^ whether targeting GOT2 is sufficient to reverse the immune suppression in this case remains to be investigated.

## Author contributions

J.B.: writing – original draft, visualization. Z.M.: writing – original draft, conceptualization. Q.Y.: writing – review & editing, supervision. All authors read and approved the final version of this manuscript.

## Funding

This work was supported by the 10.13039/501100001809National Natural Science Foundation of China (No. 31972890 to Q.Y.) and the 10.13039/501100011789Department of Science and Technology of Jilin Province, China (No. 20230101139JC to Q.Y.).

## Conflict of interests

The authors declared no competing interests.
